# Centering The Voices of People Living with Multimorbity to Develop Visual Dashboards to Facilitate Structured Medication Reviews: The Role of Patient and Public Involvement and Engagement in the DynAIRx Study

**DOI:** 10.23889/ijpds.v9i5.2842

**Published:** 2024-09-18

**Authors:** Erin McCloskey

**Affiliations:** 1 University of Liverpool

The use of Artificial Intelligence in healthcare has the potential to enhance the experiences of both patients and healthcare practitioners (Rajpurker et al., 2022). In an effort to streamline Structured Medication Reviews (SMRs) and to support medication optimization, The Artificial Intelligence (AI) for dynamic prescribing optimization and care integration in multimorbidity (DynAIRx) is a project which seeks to apply AI extracted from clinical records to improve the lives of people who are living with multimorbidity. SMRs were introduced at the primary care level to holistically support complex multimorbid patients’ health journeys and who are considered to be polypharmacy (at least five medications daily). However, there has been poor integration of health records across systems, and limited guidance on how to identify people who need yearly SMR. Without appropriate monitoring a patient’s medication, their polypharmacy may worsen (NICE, 2017); negatively impacting a patient’s overall health. Using AI and statistical approaches to predict the risk of poor outcomes, DynAIRx will produce visual dashboards for healthcare providers to use to facilitate reviews of medications for patients requiring SMRs.

A Public Advisory Steering Group, comprised of individuals with lived multimorbidity experiences or carers of people with multimorbid conditions sits at the heart of DynAIRx. The steering group is designed to ensure co-production is implemented throughout each work package. This presentation presents DynAIRx as a case study on how to develop and implement PPIE as user/partnership-centered approach to AI research in healthcare and potential implications of including the public’s perspectives on the larger product.

